# Dual RNA-Seq Enables Full-Genome Assembly of Measles Virus and Characterization of Host–Pathogen Interactions

**DOI:** 10.3390/microorganisms9071538

**Published:** 2021-07-20

**Authors:** Timokratis Karamitros, Vasiliki Pogka, Gethsimani Papadopoulou, Ourania Tsitsilonis, Maria Evangelidou, Styliani Sympardi, Andreas Mentis

**Affiliations:** 1 Public Health Laboratories, Department of Microbiology, Hellenic Pasteur Institute, 11521 Athens, Greece; vpoga@pasteur.gr (V.P.); meuagelidou@pasteur.gr (M.E.); mentis@pasteur.gr (A.M.); 2 Bioinformatics and Applied Genomics Unit, Hellenic Pasteur Institute, 11521 Athens, Greece; gesthpap@pasteur.gr; 3 Section of Animal and Human Physiology, Department of Biology, National and Kapodistrian University of Athens, 15784 Athens, Greece; rtsitsil@biol.uoa.gr; 4 1st Department of Internal Medicine, Thriasion General Hospital, 19018 Elefsis, Greece; lianasympa@hotmail.com

**Keywords:** measles virus, host response, dual RNA-seq, genome assembly, transcriptome

## Abstract

Measles virus (MeV) has a negative-sense 15 kb long RNA genome, which is generally conserved. Recent advances in high-throughput sequencing (HTS) and Dual RNA-seq allow the analysis of viral RNA genomes and the discovery of viral infection biomarkers, via the simultaneous characterization of the host transcriptome. However, these host–pathogen interactions remain largely unexplored in MeV infections. We performed untargeted Dual RNA-seq in 6 pharyngeal and 6 peripheral blood mononuclear cell (PBMCs) specimens from patients with MeV infection, as confirmed via routine real-time PCR testing. Following optimised DNase treatment of total nucleic acids, we used the pharyngeal samples to build poly-A-enriched NGS libraries. We reconstructed the viral genomes using the pharyngeal datasets and we further conducted differential expression, gene-ontology and pathways enrichment analysis to compare both the pharyngeal and the peripheral blood transcriptomes of the MeV-infected patients vs. control groups of healthy individuals. We obtained 6 MeV genotype-B3 full-genome sequences. We minutely analyzed the transcriptome of the MeV-infected pharyngeal epithelium, detecting all known viral infection biomarkers, but also revealing a functional cluster of local antiviral and inflammatory immune responses, which differ substantially from those observed in the PBMCs transcriptome. The application of Dual RNA-seq technologies in MeV-infected patients can potentially provide valuable information on the virus genome structure and the cellular innate immune responses and drive the discovery of new targets for antiviral therapy.

## 1. Introduction

Measles virus (MeV) infection causes worldwide mortality in children, as more than 100,000 fatal infections occur each year [1]. Since there is no animal reservoir of MeV, an effective and inexpensive live-attenuated vaccine is broadly available, and since there is relatively low genetic variation between the strains, measles is a candidate infection for global control and eradication [2]. However, it has been re-established in the form of major epidemics, even in industrialized countries, due to religious objections to vaccination, philosophical inhibitions and concerns about vaccination safety in general [3]. MeV belongs to the genus Morbillivirus, of the Paramyxoviridae family, and [4] has a single-stranded, negative-sense, 15 kb long RNA genome, which is generally conserved and encodes the six structural proteins, N, P, M, F, H, and L, and two nonstructural proteins, V and C (WHO, 2015) [5]. The decreased diversity of circulating MeV strains makes their differentiation more difficult. Towards a more accurate epidemiological mapping of the virus, extended genomic regions, such as the *H* and *P* genes, but also non-coding sequences, have been used [6]. Whole-genome sequencing of MeV has also shown that the revealed genetic variability was supportive in mapping MeV transmission chains [7,8].

The virus can be rapidly transmitted by dispersed respiratory droplets or through direct contact with infected objects. After approximately 10 days of incubation, the onset of high fever (>38.3 °C) is observed, accompanied by sneezing and coughing, symptoms that increase the transmissibility of the virus. The onset of a generalized maculopapular skin rash is observed 14 days post-infection, while patients are considered infectious 4 days before to 4 days after this time point, due to the higher replication rates of the virus in the respiratory tract during this period [9,10]. After its introduction to the respiratory tract at the peak of infection, contrary to other paramyxoviruses, it infects lymphocytes, dendritic cells (DCs), and macrophages [11]. The virus infects immune cells by binding to receptors distributed in a certain way, dictating which cell types and tissues will be affected [12,13]. The virus–host cell interaction is mediated by conformational changes, at first in a transmembrane glycoprotein, haemagglutinin (H), which forms tetrameric complexes and is located on the virion’s surface and subsequently in trimeric fusion viral glycoprotein complexes (F), which facilitate the fusion of the plasma membrane of the host cell with the viral envelope, leading MeV to eventually enter the cell cytoplasm [13,14]. MeV is then transported to epithelial cells of the respiratory tract and infects them by binding to the receptor nectin-4, whose cellular location is specific and can be correlated with virus-induced pathology [12,14,15]. In the epithelial cells of the lung, bronchi, and trachea, replication takes place and the newly produced viral particles are released, causing systemic disease [11] allowing transmission by respiratory aerosols [14,16]. Contrary to the typical innate immune response to viruses, in MeV infection, induction and signaling by IFN-α/β is inhibited due to the combined activity of P, C, V viral proteins, enabling extensive replication and spread of MeV [11,17]. Inflammatory response is induced in peripheral blood mononuclear cells (PBMCs) and is related to the initiation of the adaptive response essential for clearance. However, cellular responses are short-termed during rash, due to regulatory T cells’ immunosuppressive activity; therefore, clearance of viral RNA cannot be succeeded [11,18]. The existence of viral RNA and proteins in lymphoid tissue after the acute phase serves a dual purpose suppressing immune responses to new infections and promoting of maturation of Β-cell response to MeV, increasing antibody avidity, which along with CD4+ T-cell proliferation plays a crucial role in establishment of life-long protective immunity [11].

Single-analyte host biomarkers have been recruited as quantifiable indicators of inflammation; however, limited specificity and sensitivity of markers such as erythrocyte sedimentation rate and C-reactive protein remain a burden to their establishment, while probably the most useful is procalcitonin, which indicates the presence of bacterial infection in sepsis and lower respiratory tract infections [19,20]. In the past two decades, studies were almost solely based on microarray analyses, which have allowed the comprehensive analysis of host’s response to infections and host transcriptomic signatures in PBMCs have been shown to distinguish bacterial from viral infections [21,22,23]. However, the power of the unbiased RNA-seq transcriptomics has been applied to the study of the interactions between the host and the pathogen transcriptomes at the same time (dual RNA-seq), and it seems that the transcriptomics profiling of the host PBMCs can be a useful tool in characterizing respiratory infections [24,25]. The untargeted dual RNA-seq approach was used for the qualitative detection of respiratory RNA viruses and the simultaneous quantification of the airway transcriptome directly on the pharyngeal epithelium [26]. Additionally, nasal airway gene expression profiles can distinguish disease sub-phenotypes [27], which further points out the role of the epithelial transcriptome as a potential source of valuable biomarkers.

## 2. Materials and Methods

**Figure 1:**
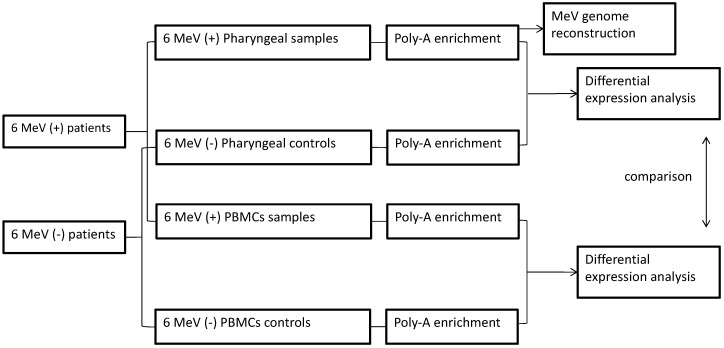
Study design and pairwise comparisons. Total human transcriptome analysis was performed in 24 samples in total. Pharyngeal and PBMCs samples were analyzed from 6 MeV (+) and 6 MeV (−) patients. Differential expression analysis was performed between MeV (+) and MeV (−) samples. Each of the 6 MeV (+) pharyngeal samples was also analyzed for the reconstruction of the viral genome.

We retrospectively analyzed total nucleic acids (stored at −80 °C) extracted from pharyngeal swabs and PBMCs of 6 fully anonymized patients with MeV genotype B3 infection, as confirmed via routine real-time PCR testing for viral infections. In parallel, two sets of nucleic acid samples (pharyngeal and PBMCs) were analysed from 6 clinically and laboratory healthy individuals, as controls (Figure 1). Following DNase treatment (Turbo DNase, ThermoFisher Scientific, Waltham, MA, USA) of the nucleic acids, the RNA-seq libraries were prepared for sequencing using theTruseq V2 kit (Illumina, San Diego, CA, USA), with poly-A RNA enrichment. We performed untargeted dual RNA-seq on high-throughput Next-Seq Illumina flow cells, which yielded 1,026,402,011 million single-ended, 75 bp long reads in total.

The consensus genomes were generated after mapping alignment of the reads to the reference strain MeVs/London.GBR/3.14 (genotype B3-KT732219.1) using Bowtie2 [28]. Samtools were used for all file transformations and for calling the variations from the mapping alignments [29]. The genotyping results were confirmed and quality-controlled by visual inspection of the mapping alignments using IGV [30]. The RNA-seq analysis was performed using Kallisto [31] and the human reference transcriptome v.GRCh38.rel79, in order to calculate the abundances of the transcripts. Sleuth package [32] and R-base functions were used to interpret and visualize the RNA-seq analysis results. Gene Ontology (GO) and KEGG pathway enrichment analysis was performed using the differentially expressed genes (absolute fold of change ≥ 2, q < 0.01).

## 3. Results

Each of the 6 MeV (+) pharyngeal samples was analyzed twice, for both the reconstruction of the viral genome and the characterization of the host transcriptome. Poly-A enrichment yielded an average of 2.38 × 10^7^ reads per library, while 2.29% of these reads were successfully mapped on the viral genome on average. The average genome coverage and depth was 99.87% and 1330.2×, respectively (Table 1). The reconstruction of the genomes was complete, with 100% of the viral genome being covered in all cases, with the exception of one sample (MeV2) where 99.25% of the MeV genome was covered. Apart from the qualitative detection, the reconstructed genomes allowed the accurate genotyping of the samples using the consensus sequence as a BLAST query.

**Figure 2:**
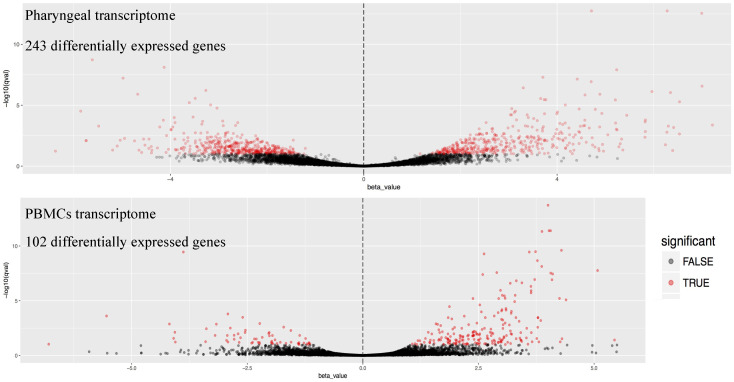
Volcano plots of the differentially expressed genes found in the Pharyngeal (top) and the PBMCs (bottom) transcriptome. Statistically significantly differentially expressed genes are shown in red.

Dual RNA-seq analysis revealed the transcriptomic signatures of both pharyngeal epithelium and PBMCs of the patient specimens. By comparison to uninfected samples, we were able to identify all the differentially expressed genes (Supplementary Tables S1 and S2) and to characterize the profile of the biological functions involved in each transcriptomic response. The differentially expressed genes in the pharyngeal transcriptome were more than double in total, compared to those in PBMCs (243 vs. 102 genes). The latter were mainly upregulated upon MeV infection, −86 upregulated vs. 16 downregulated genes-, while the upregulated and downregulated genes in the pharyngeal transcriptome were 161 and 41, respectively (Figure 2). Moreover, by analyzing and clustering the GO terms linked to these genes, we revealed that biological functions were mainly associated with the response to viral infection in the case of the pharyngeal transcriptome, while PBMCs transcriptomic signatures indicated activation of cell proliferation, which mirrors a general immunological response to infection. This finding indicates that the pharyngeal transcriptome might represent a better source of host response biomarkers for identification of viral infections compared to the PBMCs transcriptome.

**Figure 3:**
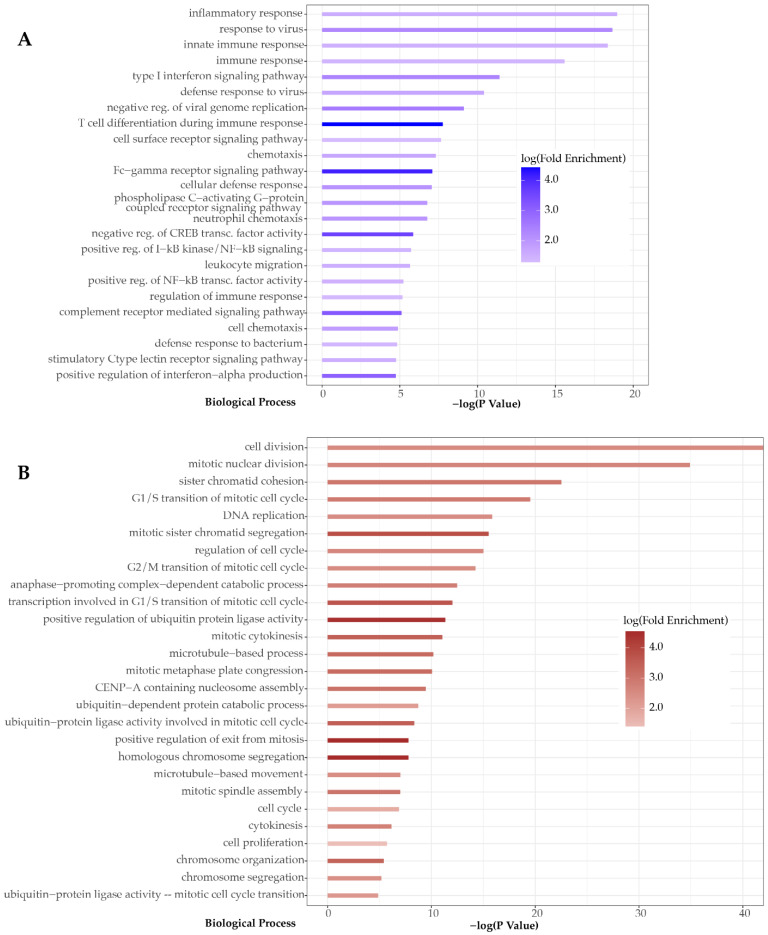
Significantly enriched (*p* < 0.05) biological processes based on the Gene Ontology (GO) enrichment analysis of the differentially expressed genes in the pharynx (**A**) and in the PBMCs (**B**).

Analysis of the pharyngeal epithelium transcriptome revealed several key genes known to be involved in the host immune responses. Among all differentially expressed genes, two upregulated genes, *RAD2* and *OASL*, known to be rapidly induced in response to viral infection, were observed [26,33]. The *IFIH1* (*MDA-5*) gene, which has been shown to trigger the release of interferons (IFNs), was also upregulated [34]. Additionally, we observed upregulation of several IFN-induced genes (*IFIT3*, *IFIT2*, *IFIT1*, *IFITM3*, *IFI44*), as well as of the *IVNS1ABP* (*NS-1*) gene, which encodes Influenza virus NS1A-binding protein, an antagonist of IFNs I and III [35]. Overexpression was also observed in the case of *TNFAIP3*/*A20*, a gene that encodes a zinc finger protein, which acts as inhibitor of both NFκB activation and TNF-mediated apoptosis [36]. Innate immunity activation was also depicted by the increased expression of *CLEC7A* (*DECTIN 1*), *CLEC4E*, and *TNFAIP6* genes [37,38]. Finally, overexpression of chemokines CXCL1 and CXCL2 indicates presence of inflammation in the pharynx and is considered a transcriptomic signature, as it leads to the induction of NLRP3 inflammasome in macrophages [39]. The *AIM2* gene was upregulated, allowing the assumption that dsDNA-dependent AIM2-inflammasome activation is successful, leading to AIM2-mediated IL-1β and IL-18 production [40] (Figure 3).

Contrary to the pharyngeal transcriptome, the host response signatures in the PBMCs were not directly linked to host response to viral infections. Differentially expressed genes, 86 in total, were linked to cell cycle-related processes. According to the Gene Ontology (GO) enrichment analysis using the differentially expressed genes, *CDC45*, *MCM2*, *GINS2*, *ORC1*, and *CDC25A* are involved in DNA replication and more specifically in the regulation of G1/S transition of mitotic cell cycle and of DNA-dependent DNA replication initiation. The products of the abovementioned genes mediate DNA replication, as CDC25 and MCM2, along with MCM3-7 and GINS, form CMG complex, which acts as a replicative helicase [41]. MCM2-7 subunits form a long channel in order for double-stranded DNA to pass through and such action requires CDC6, CDT1, and Origin Recognition Complex (ORC) proteins, two of which were upregulated (Figure 3).

Moreover, GO analysis revealed that among the differentially genes there is a group related to cell division (*CDC20*, *PTTG1*, *CDC25A*, *CCNB1*, *CCNB12*), particularly linked to regulation of cell cycle checkpoints (*CCNB1*, *E2F2*, *CCNB2*, *PLK1*, *PKMYT1*, *CDC25A*). “Cell division cycle 25 A”, a phosphatase encoded by the *CDC25A* gene, plays an important role in cell division, regulating the activities of cyclin-dependent kinases (CDKs) in a positive way, removing phosphorylation that acts as inhibitor [42]. Specifically, CDC25A activates the cyclin E-CDK2 complex by dephosphorylating two residues on CDK2 and the complex phosphorylates Rb, which, in its dephosphorylated form, acts as a suppressor of E2F transcription activity [42]. E2F (E2F1, E2F2, E2F3) transcriptional activators, upregulated in our study, are able to induce CDC25A transcription by binding to the promoter of the gene and subsequent activation of CDC25A promotes G1/S progression [42]. Additionally, CDC25A interacts with cyclin-dependent kinase (CDK1), activating it in order to interact with cyclin B, which is encoded by the *CCNB* gene [43,44], also upregulated in our study. PLK1, a mitotic inducer overexpressed based on the current analysis, accumulates affecting activation of CDK1 [45]. Completion of mitosis is promoted by a negative regulator of the CDK1/CycB complex, PKMYT1, upregulated in our study. PBMCs GO analysis also revealed upregulation of the *PTTG1* gene, encoding a protein whose active role is to maintain genomic stability during mitosis, by controlling sister chromatids’ segregation [46].

## 4. Discussion

Unbiased—dual RNA-seq transcriptomics can decipher the interactions between the host and pathogen genomes and transcriptomes at the same time. The aim of this study was the simultaneous reconstruction of the virus genome and the detailed characterization of the pharyngeal and the PBMCs’ transcriptome of MeV-infected patients. Differential expression RNA-seq analysis of the pharyngeal epithelium depicted upregulation of *RSAD2*, *OASL*, and *IFIT2* IFN-stimulated genes (ISGs) encoding intracellular proteins. Elevated levels of these proteins are correlated with response to viral infection [47,48]. Specifically, detection of MeV by RIG-1 and MDA-5 pattern-recognition receptors (PRRs) and activation of these receptors results in the production of IFN-β and subsequent induction of the abovementioned ISGs [47]. OASL and IFIT2 act as effectors, preventing replication of the virus and viperin, a protein encoded by *RSAD2*, acting as inhibitor of MeV release in infected cells [49]. Viperin together with IFITM2 and IFITM3 are reported to have antiviral activity, as processes of binding, entry, and uncoating of the nucleocapsid are considered as targets of IFITM proteins and are inhibited in case of, for example, West Nile and Dengue virus infections [50,51]. Moreover, *OASL* and *IFIT2* together with *CXCL10* form a group of genes whose mRNAs levels can be considered as biomarkers, predicting respiratory virus infection with an accuracy of 97% [48]. CXCL10 is a chemokine that acts as a chemotactic factor attracting T cells to the site of the infection, and its levels are highly associated with the presence of viral infection [48,52]. In general, inhibition of induction and signaling by IFN-α/β [24] is not reflected in our results, as we observed many IFN-induced upregulated genes (*IFIT3*, *IFIT2*, *IFIT1*, *IFITM3*, *IFI44*). This can be explained by the elevated levels of *IFIH1* gene, encoding MDA-5 PRR, as well as by the action of the cytoplasmic helicase induced by IFN, encoded by the upregulated *DDX60* gene. The helicase acts as guardian for viral RNA degradation and RIG-I activation in response to viral RNA and DNA [53]. Particularly, it acts upstream of RIG-I in the innate immune response and is involved in RIG-I-dependent type I IFN production, activating ligand-dependent RIG-I signaling [53]. Viral RNA binds to RIG-I, causing conformational changes, enabling its interaction with MAVS, subsequently promoting translocation of IRF-3 and NF-κB required for INF-β gene transcription [54]. It should be mentioned that except from MDA-5 and RIG-1 activation, TLR3 signaling can also induce IFN production in conventional DCs, although the receptor is not overexpressed in our study, neither in the PBMCs nor in the pharyngeal epithelium [55]. Finally, robust innate immune response via IFN signaling is induced in a cell-type specific way, as cell lines behave in a different way as in vitro studies report. For example, microarray analysis of the epithelial 293SLAM cell line indicated that IFN-I signaling was not blocked, contrary to the findings concerning lymphoid COBL-a cell line [56]. Specifically, early in the infection (6 h post-infection) the activation of IRF3 promotes IFN-I and subsequently ISGs production. On the other hand, suppression of the IFN-I signaling pathway in COBL-a is typical of MeV infection [56]. Of note is the study of Donohue et al. regarding MeV quasispecies adaptation to different cell types [57]. During adaptation of the virus, multiple variants aggregate in a specific region of the *P* (phosphoprotein) gene, affecting downstream RNA editing, resulting in the production of the IFN-antagonist, V. Ιn the lymphocytic cell line Granta-519, MeV variants negatively affect expression of functional V protein, while in the epithelial cell line H358, no variants are reported proximal to the editing site, V’s production is not affected, and the protein can act as a negative regulator of IFN-I signaling [57].

Despite the abovementioned, IFN-I transcripts were not detected in our study, a finding in agreement with the literature. We report that the *IVNS1ABP* (*NS-1*) gene, found overexpressed in the current study, encodes Influenza virus NS1A-binding protein and acts as an antagonist of host type I and III IFN production and signaling [35]. Moreover, it is known to play a role in apoptosis inhibition, DCs maturation suppression as well as control of protein stability, and acts as regulator of transcription of host cell mRNAs [35]. Like other known pathogens (e.g., Influenza virus), MeV carries components that mimic regulatory elements of the host cells, enabling virus to intervene in essential processes and NS-1 protein is one of those key molecules, as it suppresses antiviral genes’ expression acting as a histone mimic, as it possesses a H3-like sequence [58,59].

From another set of known key components (*STAT1*, *STAT2*, *p53*, *IFIH1*), considered as targets of MeV [36], only *IFIH1* gene was upregulated in our study. The cytoplasmic receptor encoded by this gene binds to dsRNA and undergoes structural rearrangements, triggering the release of proinflammatory cytokines, particularly IFNs by immune cells, thus inducing apoptosis of virus-infected cells [34,60]. However, V inhibitor protein of MeV specifically binds to IFIH1, unfolds its ATPase domain inhibiting ATPase activity and cellular aggregation of IFIH1, disrupting activation of the downstream signaling cascade [60].

According to the results of the GO enrichment analysis, two members of the protein family TNFAIPs (Tumor necrosis factor alpha-induced proteins) are upregulated, TNFAIP3 and TNFAIP6. In general, members of this protein family differ more than 75% at the level of amino acid residues, indicating different biological function and their gene expression is induced by TNF-α [61]. The *TNFAIP3*/*A20* gene encodes a zinc finger protein acting as a ubiquitin modification enzyme, which plays a role as negative-feedback regulator of the NF-κB signaling pathway and as mediator of TNF apoptosis [61,62]. TNFAIP3 downregulates the activity of IRF-3, a key transcription factor for the induction of IFN-γ [36]. During MeV infection, viral P protein activates the expression of the ubiquitin modifier TNFAIP3, which interacts with TRAF6-binding protein, TAX1BP1, forming a complex that acts as TRAF6 inhibitor by preventing E3 ligase TRAF6 polyubiquitination [63], thus blocking the TLR4-mediated proinflammatory signaling [63]. The second member of the family found to be overexpressed in our study is TNFAIP6, known as TSG-6, a secreted multifunctional protein, produced at sites of inflammation, which acts as mediator of tissue remodeling and anti-inflammatory responses, mainly by regulating chemokines [61,64,65]. Human TNFAIP6 has a Link module (Link_TSG6) which binds to CXC- and CC-chemokines inhibiting neutrophile migration and their influx to inflammatory sites [64].

In our study, overexpression of *CLEC7A* (*DECTIN 1*), a component of innate immune response, was also observed. It is known that the C-type lectin dectin-1 is a robust inducer of Th1 and/or Th17 responses. CLEC7A inhibits TLR4 signaling, downstream inflammation, and chemokines secretion, acting as a mixed blessing on the regulation of inflammation [38,66]. Except from TNFAIP6 upregulation, another component of the innate immune response and inflammation, CLEC4E, was upregulated, and this finding is in agreement with previous studies reporting upregulation of nine signaling molecules (CEBPB, HP, SAA1, CLEC4E, PTX3, TNFAIP6, SERPINE1, interleukin-6 (IL-6), IL1RN) during the acute phase of the lethal Ebola Virus infection [37]. It should be taken into account that CLEC4E appears to have a dual role in inflammatory condition, by promoting production of pro- and anti-inflammatory cytokines based on PRRs and their ligands that mediate immune response in each case [38,67]. Inflammasome is activated during MeV infection, a fact supported by our findings that report upregulated expression of chemokines CXCL1 and CXCL2, which results in activation of NLRP3 inflammasome in macrophages, an effect mediated by their interaction with the related receptor CXCR2 [39]. CXCL1 is a ligand that acts as a chemoattractant recruiting immune cells, like monocytes, neutrophiles and T-lymphocytes [39,68]. Moreover, CXCR2 binding to CXCL2, the structurally related GRO family chemokine produced by macrophages, contributes to lung injury during viral infection, an event crucial for the establishment of pro-inflammatory conditions associated with respiratory disorders [69]. These findings are in accordance with the observation that the pharyngeal transcriptomic signature in our dataset was more informative with regard to detection of viral infections.

The majority of overexpressed genes in PBMCs encode protein molecules that are activated during DNA replication (*CDC45*, *MCM2*, *ORC1, CDC25A), cell division (CDC20, PTTG1, CDC25A, CCNB1, CCNB12*) or act as regulators in cell cycle checkpoints (*CCNB1*, *E2F2*, *CCNB2*, *PLK1*, *PKMYT1*, *CDC25A*). DNA replication and mitosis entry and exit are strictly regulated by a complex feedback system, which depends mainly on phosphorylation and dephosphorylation events by a number of kinases and phosphatases, respectively [43]. Regarding DNA replication processes, the overexpression of CDC45, MCM2, and ORC1 proteins depicted in our results is expected, considering their association with events taking place at the G1, S, and G2 cell cycle phases [70,71]. In order for DNA replication in eukaryotes to be initiated in the S phase, protein–protein interactions take place, as MCM2-7 helicase is loaded onto double-stranded DNA and activated by GINS-CDC45 [72,73]. CDC45 acts as a limiting factor for replication initiation and the activation of CMG helicase in humans [74]. Moreover, studies using HeLa cells report that MCM2-7 complex molecules are 10–50-fold more than CDC45 molecules [75]. Upregulation of GINS, ORC1-CDT1, and MCM2 in our study indicates a possible association between complexes since CDT1 and MCM form a complex which stabilizes MCM subunits during conformation of the MCM ring [41].

The G2-to-M transition and exit from mitosis following cell division are regulated by switch-like reactions driven by protein molecules subjected to different states of phosphorylation. Cell division cycle 25 A (CDC25A) protein phosphatase plays an important role as it has dual specificity, in activation of CDKs [43,76]. Specifically, it dephosphorylates CDK2, CDK4, and CDK6, which, in turn, during cell cycle transition from G1 to S, phosphorylate Rb, resulting in dissociation of E2Fs transcription factors from Rb and subsequently their activation [76]. Their essential role in cell cycle processes offers an explanation to the upregulation of CDC5A and E2Fs, reported in our study. CDC25A interacts with another CDK1, removing the inhibitory phosphorylations, resulting in its activation, enabling its subsequent interaction with cyclin B (CycB) [43,44]. This protein, encoded by the *CCNB* gene, also upregulated in this study, dynamically alters its subcellular localization from the cytoplasm to the nucleus, interacts with CDK1, forming a complex which mediates cell cycle progression, by phosphorylating substrate targets [75]. Activation of CDK1 is also affected by accumulation of molecules which induce mitosis, such as PLK1, found to be upregulated in our study. The activity of CDK1/CycB complex during the G2/M transition is balanced by feedback mechanisms, the already mentioned dephosphorylation by Cdc25A [43], and degradation of CycB and rephosphorylation by PKMYT1 [43,77,78]. Protein kinase, membrane-associated tyrosine/threonine kinase of the Wee family, is encoded by the *PKMYT1* gene, upregulated in our study, and acts as negative regulator in the G2/M phase, promoting completion of mitosis, as it phosphorylates Tyr14/Tyr15 inactivating the CDK1/CycB complex [79]. Another gene, *PTTG1*, the active role of which in mitosis was revealed by GO enrichment analysis in PBMCs, is a proto-oncogene encoding a protein responsible for maintenance of genomic stability during mitosis [46]. *PTTG1* is upregulated during cell cycle, specifically at the G2/M phase, and actively controls separation of sister chromatids [46]. Moreover, PTTG1 with its SH-3 domain interacts with multiple signaling pathways and is involved in activation of growth factor pathways by mediating activity of Src kinase [46].

In general, our results agree with what is reported on genetic reconstruction of immune cell pool after MeV infection. Infection of immune cells takes place after binding of viral hemagglutinin (H) to cellular receptors CD150, resulting in depletion of specific subsets of B and T lymphocytes [44,80]. In recent studies, not only loss of expanded B and T memory clones is observed, but also reduction of antibody production and repertoire, due to loss of long-lived plasma B-cell subset and incomplete recovery of pre-infection diversity [80,81,82]. As successful regeneration of naïve B lymphocytes takes place approximately four weeks after infection, no change in frequencies of the lymphocyte population is observed [83].

The major conclusion drawn from these observations is that massive infection of lymphocytes and depletion of effector cells and memory clones results in damaged adaptive responses and eventually induction of immune suppression [83,84]. Specifically, certain types of B and T lymphocytes are depleted, while the naive population is not influenced and a major consequence of this repertoire alteration is increased susceptibility to secondary infections, due to abolishment of immunological memory [83].

## Figures and Tables

**Table 1:** MeV genome alignment statistics based on reference genome KT732219.1.

Sample	Enrichment Method	Total Reads	Total MeV Genome Reads	MeV Reads (%)	Average Depth (×)	MeV Genome Coverage (%)
Pharg_MeV1	poly-A	34,370,942	405,851	1.18	1953.95	100.00
Pharg_MeV2	poly-A	34,817,617	282,092	0.81	1357.61	99.25
Pharg_MeV3	poly-A	28,188,497	90,601	0.32	436.01	100.00
Pharg_MeV4	poly-A	29,716,507	59,901	0.20	288.41	100.00
Pharg_MeV5	poly-A	12,783,974	620,934	4.86	2990.41	100.00
Pharg_MeV6	poly-A	3,126,258	198,309	6.34	955.13	100.00

## Data Availability

Data presented in Supplementary Material.

## Supplementary Materials

The following are available online at https://www.mdpi.com/article/10.3390/microorganisms9071538/s1; Supplementary Table S1: Differential Expressed genes in the pharyngeal epithelium; Table S2: Differential Expressed genes in PBMCs.
