# Supplementary material for: Dual RNA-Seq Enables Full-Genome Assembly of Measles Virus and Characterization of Host–Pathogen Interactions
Source: Microorganisms. 2021 Jul 20;9(7):1538. doi: 10.3390/microorganisms9071538 (PMC8303570; doi:10.3390/microorganisms9071538)
Supplement: Supplementary file 1 [file microorganisms-09-01538-s001.zip › microorganisms-1288382-supplementary.pdf]

microorganisms

MDPI

Supplementary Material

Dual-RNAseq enables full genome assembly of measles virus and characterization of host-pathogen interactions

Timokratis Karamitros [1,2,*], Vasiliki Pogka [1,†], Gethsimani Papadopoulou [2,3,†], Ourania Tsitsilonis [3], Maria Evangelidou [1], Styliani Sympardi [4] and Andreas Mentis [1]

1 Public Health Laboratories, Department of Microbiology, Hellenic Pasteur Institute, 11521 Athens, Greece; vpoga@pasteur.gr (V.P.); meuagelidou@pasteur.gr (M.E.); mentis@pasteur.gr (A.M.)
2 Bioinformatics and Applied Genomics Unit, Hellenic Pasteur Institute, 11521 Athens, Greece; gesthpap@pasteur.gr (G.P)
3 Section of Animal and Human Physiology, Department of Biology, National and Kapodistrian University of Athens, 15784 Athens, Greece; rtsitsil@biol.uoa.gr (O.T.)
4 1st Department of Internal Medicine, Thriasion General Hospital, 19018 Elefsis, Greece; lianasympa@hotmail.com (S.S)
* Correspondence: tkaram@pasteur.gr; Tel.: +30-2106478871
† Equally contributed

Supplementary Table S1.

Differential Expressed genes in the pharyngeal epithelium

geneName	betaValue	pValue	qValue
IFIT1	7.213	2.99E-06	4.24E-04
CLEC4E	6.998	3.33E-10	2.71E-07
MCEMP1	6.992	9.51E-17	2.83E-13
FCGR1A	6.532	1.41E-08	5.24E-06
IFIT2	6.531	3.04E-05	2.30E-03
FCGR1B	6.410	5.98E-06	6.76E-04
CCL3L3	6.345	1.53E-09	9.12E-07
FCER1G	6.300	4.05E-06	5.17E-04
DSG3	6.278	3.78E-17	1.81E-13
IFIT3	6.270	1.85E-05	1.65E-03
DSC2	5.961	1.18E-09	7.53E-07
CLEC2B	5.827	8.74E-07	1.63E-04
OASL	5.825	1.57E-05	1.45E-03
TNFAIP6	5.820	1.40E-06	2.31E-04
HERC5	5.818	7.16E-05	4.21E-03
C3AR1	5.441	3.15E-07	6.70E-05
C1orf162	5.334	1.22E-04	6.03E-03
SPRR2F	5.241	1.08E-05	1.06E-03
LTF	5.232	8.28E-12	1.23E-08
CLEC4D	5.224	9.05E-06	9.30E-04
LY96	5.210	1.92E-06	2.90E-04
BCL2A1	5.206	9.39E-09	3.65E-06
IFI6	5.049	3.95E-05	2.71E-03
MNDA	4.925	1.89E-07	4.70E-05
HESX1	4.885	2.38E-09	1.25E-06
CXorf21	4.880	5.10E-06	6.07E-04
AQP9	4.791	7.53E-08	2.17E-05
ZNF438	4.724	8.40E-05	4.72E-03
BPIFA1	4.711	4.05E-17	1.81E-13
DDX60L	4.705	1.30E-10	1.16E-07
RSAD2	4.665	6.83E-06	7.44E-04
CLEC4A	4.577	2.88E-09	1.43E-06
FCGR3B	4.573	9.45E-07	1.69E-04
CCRL2	4.572	3.38E-06	4.65E-04
LMNB1	4.544	3.76E-06	4.94E-04
CCL4L2	4.503	8.56E-05	4.78E-03
EVI2A	4.482	4.37E-06	5.34E-04

MUC2	4.467	1.91E-05	1.69E-03
GIMAP4	4.456	6.80E-08	2.03E-05
IFITM3	4.447	1.57E-05	1.45E-03
CARD16	4.416	7.22E-11	7.17E-08
SPRR2G	4.377	3.82E-05	2.68E-03
GPR84	4.339	6.98E-05	4.18E-03
SRGN	4.257	3.72E-05	2.66E-03
GPR65	4.245	6.65E-06	7.34E-04
LILRB2	4.173	9.08E-05	4.98E-03
CD53	4.169	2.87E-08	9.49E-06
CST7	4.167	1.86E-06	2.87E-04
MCTP1	4.124	2.04E-04	8.31E-03
OLR1	4.117	1.51E-04	6.82E-03
IFITM2	4.116	2.67E-06	3.85E-04
FFAR2	4.103	2.58E-05	2.08E-03
FYB1	4.077	1.68E-05	1.54E-03
FCAR	4.057	2.09E-06	3.11E-04
GBP1P1	4.051	1.25E-04	6.05E-03
P2RY14	4.038	2.77E-05	2.16E-03
EVI2B	4.014	9.76E-05	5.22E-03
NFIL3	4.008	1.37E-07	3.59E-05
SELL	3.994	2.06E-07	4.98E-05
CD177	3.994	9.58E-06	9.62E-04
NTNG2	3.968	2.71E-05	2.14E-03
AOAH	3.933	5.91E-05	3.69E-03
FPR2	3.899	1.30E-04	6.17E-03
AIM2	3.791	2.07E-04	8.34E-03
CASP1	3.767	8.28E-09	3.49E-06
NBN	3.747	1.59E-04	7.04E-03
NSMAF	3.740	2.12E-07	4.98E-05
CMPK2	3.739	2.07E-04	8.34E-03
FCGR2A	3.737	1.78E-04	7.52E-03
S100A12	3.733	8.61E-09	3.49E-06
FPR1	3.715	4.83E-06	5.83E-04
SERPINB3	3.705	3.96E-11	5.06E-08
DHRS12	3.698	1.39E-05	1.32E-03
ALOX5AP	3.696	7.96E-06	8.46E-04
FCGR3A	3.669	1.21E-04	6.03E-03
IGSF6	3.659	6.34E-09	2.83E-06
CCDC71L	3.629	8.97E-05	4.98E-03
ADGRG3	3.623	1.44E-04	6.74E-03
SERPINB7	3.609	5.44E-05	3.52E-03

CXCR2	3.602	1.65E-04	7.22E-03
TMEM71	3.590	1.05E-06	1.84E-04
NCF2	3.586	1.14E-05	1.10E-03
IFI44	3.526	1.01E-04	5.30E-03
SDS	3.520	7.03E-05	4.19E-03
TNFSF13B	3.516	6.38E-07	1.24E-04
DDX60	3.484	5.31E-06	6.12E-04
SERPING1	3.471	7.45E-05	4.35E-03
MS4A6A	3.470	2.12E-04	8.42E-03
CLEC7A	3.461	5.39E-05	3.52E-03
PLAUR	3.409	9.09E-05	4.98E-03
ANKRD22	3.404	5.04E-07	1.02E-04
TYROBP	3.392	4.32E-06	5.34E-04
DYNAP	3.388	5.66E-05	3.62E-03
PNRC1	3.380	2.76E-07	6.15E-05
RARRES1	3.370	6.77E-05	4.08E-03
TMEM140	3.342	3.76E-06	4.94E-04
TLR4	3.331	2.28E-04	8.85E-03
STATH	3.300	5.01E-10	3.73E-07
LRRK2	3.256	2.40E-04	9.07E-03
IFIH1	3.229	5.91E-05	3.69E-03
MS4A7	3.205	8.24E-05	4.69E-03
PLEK	3.204	1.05E-04	5.44E-03
ACSL1	3.203	1.27E-05	1.22E-03
PILRA	3.160	8.41E-07	1.60E-04
CYSLTR1	3.138	1.12E-07	3.07E-05
NT5C3A	3.114	1.52E-07	3.89E-05
RGS18	3.114	2.35E-04	8.94E-03
GBP3	3.085	4.34E-06	5.34E-04
ASPRV1	3.068	1.72E-04	7.39E-03
SERPINA1	3.062	6.22E-08	1.92E-05
ARL5B	3.058	2.82E-07	6.15E-05
SULT1B1	3.047	6.01E-05	3.71E-03
LYRM1	3.035	2.65E-07	6.08E-05
LYSMD2	2.985	6.62E-06	7.34E-04
TNFSF10	2.946	1.44E-04	6.74E-03
NAIP	2.910	8.33E-05	4.71E-03
CREM	2.909	2.85E-05	2.20E-03
SNX20	2.889	2.71E-05	2.14E-03
FAS	2.862	5.12E-05	3.36E-03
C19orf38	2.847	2.58E-04	9.59E-03
TXNDC12	2.785	1.24E-04	6.04E-03

TMEM123	2.745	4.62E-05	3.10E-03
NINJ1	2.739	4.34E-05	2.94E-03
FBXL5	2.738	1.51E-04	6.82E-03
VAMP5	2.713	3.89E-05	2.70E-03
PGS1	2.710	1.45E-04	6.76E-03
KCNJ15	2.707	3.43E-05	2.53E-03
GOLGA4	2.696	7.77E-05	4.51E-03
CXCL2	2.684	1.49E-04	6.82E-03
CREG1	2.667	7.63E-06	8.22E-04
LYST	2.667	1.17E-04	5.99E-03
FAM49B	2.661	1.58E-05	1.45E-03
RAB20	2.651	8.22E-06	8.64E-04
PTEN	2.589	1.81E-04	7.61E-03
PLA2G7	2.566	2.01E-04	8.27E-03
TRIM38	2.544	2.59E-05	2.08E-03
RIPK2	2.542	2.35E-05	1.97E-03
SOD2	2.530	1.63E-06	2.65E-04
HERC6	2.512	2.57E-04	9.59E-03
FNDC3B	2.491	1.21E-04	6.03E-03
S100A7	2.485	1.76E-04	7.49E-03
TNFAIP3	2.460	3.62E-05	2.63E-03
DDIT3	2.429	1.07E-04	5.52E-03
BLOC1S2	2.423	3.07E-05	2.31E-03
JMJD1C	2.397	2.78E-05	2.16E-03
TDP2	2.391	3.18E-05	2.36E-03
CASP4	2.388	8.36E-06	8.69E-04
DHX29	2.380	1.57E-04	7.00E-03
RTP4	2.375	3.77E-05	2.67E-03
ATG3	2.327	3.69E-05	2.66E-03
HACD4	2.311	1.25E-04	6.05E-03
USP15	2.280	9.54E-05	5.13E-03
TXNIP	2.279	1.47E-04	6.82E-03
IVNS1ABP	2.204	3.97E-05	2.71E-03
AKAP9	2.201	1.44E-04	6.74E-03
VASP	2.190	9.17E-05	4.99E-03
EIF1B	2.151	1.70E-04	7.39E-03
CIR1	2.115	2.34E-04	8.92E-03
CLTC	2.110	1.84E-04	7.68E-03
SH3GLB1	2.110	1.23E-04	6.04E-03
RILPL2	2.005	1.51E-04	6.82E-03
PLXNA3	-1.458	1.71E-04	7.39E-03
TUFT1	-2.093	2.34E-04	8.92E-03

PDLIM1	-2.190	1.62E-04	7.12E-03
MTRNR2L4	-2.194	1.03E-04	5.33E-03
ACTN4	-2.213	1.27E-04	6.08E-03
ARL4C	-2.237	4.92E-05	3.26E-03
CARD11	-2.262	2.33E-04	8.92E-03
PIK3IP1	-2.421	2.68E-04	9.89E-03
MT-ND1	-2.433	1.72E-04	7.39E-03
WFDC2	-2.516	3.84E-05	2.68E-03
CLUH	-2.547	8.17E-05	4.68E-03
MTATP6P1	-2.607	1.68E-06	2.67E-04
SIRT3	-2.619	5.68E-05	3.62E-03
TACSTD2	-2.651	3.30E-06	4.60E-04
MT-CO3	-2.698	2.54E-05	2.08E-03
MT-CO1	-2.734	1.02E-04	5.31E-03
FBXW4	-2.770	2.41E-04	9.07E-03
ITPR3	-2.806	2.02E-04	8.30E-03
DZIP1L	-2.808	1.48E-04	6.82E-03
MUC4	-2.809	1.82E-05	1.64E-03
ZBTB4	-2.848	2.20E-05	1.89E-03
LCN2	-2.857	2.69E-04	9.89E-03
LGALS3	-2.859	3.57E-05	2.62E-03
CXCL3	-3.444	1.29E-04	5.73E-03
CREG2	-3.579	1.30E-04	5.76E-03
LYST	-3.713	1.31E-04	5.78E-03
FAM49B	-3.847	1.31E-04	5.81E-03
RAB21	-3.982	1.32E-04	5.83E-03
PTEN	-4.116	1.33E-04	5.86E-03
PLA2G8	-4.250	1.34E-04	5.89E-03
TRIM39	-4.384	1.34E-04	5.91E-03
RIPK3	-4.519	1.35E-04	5.94E-03
SOD3	-4.653	1.36E-04	5.96E-03
HERC7	-4.787	1.37E-04	5.99E-03
FNDC3B	-4.922	1.37E-04	6.01E-03
S100A8	-5.056	1.38E-04	6.04E-03
TNFAIP4	-5.190	1.39E-04	6.06E-03
DDIT4	-5.325	1.40E-04	6.09E-03
BLOC1S3	-5.459	1.40E-04	6.11E-03
JMJD1C	-5.593	1.41E-04	6.14E-03
TDP3	-5.728	1.42E-04	6.17E-03
CASP5	-5.862	1.43E-04	6.19E-03
DHX30	-5.996	1.43E-04	6.22E-03
RTP5	-6.131	1.44E-04	6.24E-03

ATG4	-6.265	1.45E-04	6.27E-03
HACD5	-6.399	1.46E-04	6.29E-03
USP16	-6.533	1.46E-04	6.32E-03
TXNIP	-6.668	1.47E-04	6.34E-03
IVNS1ABP	-6.802	1.48E-04	6.37E-03
AKAP10	-6.936	1.49E-04	6.40E-03
VASP	-7.071	1.49E-04	6.42E-03
EIF1B	-7.205	1.50E-04	6.45E-03
CIR2	-7.339	1.51E-04	6.47E-03
CLTC	-7.474	1.52E-04	6.50E-03
SH3GLB2	-7.608	1.52E-04	6.52E-03
RILPL3	-7.742	1.53E-04	6.55E-03
PLXNA4	-7.877	1.54E-04	6.57E-03
TUFT2	-8.011	1.55E-04	6.60E-03
PDLIM2	-8.145	1.55E-04	6.62E-03
MTRNR2L5	-8.279	1.56E-04	6.65E-03
ACTN5	-8.414	1.57E-04	6.68E-03
ARL4C	-8.548	1.58E-04	6.70E-03
CARD12	-8.682	1.58E-04	6.73E-03
PIK3IP2	-8.817	1.59E-04	6.75E-03
MT-ND2	-8.951	1.60E-04	6.78E-03
WFDC3	-9.085	1.61E-04	6.80E-03
CLUH	-9.220	1.61E-04	6.83E-03
MTATP6P2	-9.354	1.62E-04	6.85E-03
SIRT4	-9.488	1.63E-04	6.88E-03
TACSTD3	-9.623	1.64E-04	6.91E-03
MT-CO1	-9.757	1.64E-04	6.93E-03
MT-CO3	-9.891	1.65E-04	6.96E-03
FBXW5	-10.026	1.66E-04	6.98E-03
ITPR4	-10.160	1.67E-04	7.01E-03
DZIP1L	-10.294	1.67E-04	7.03E-03
MUC5	-10.428	1.68E-04	7.06E-03
ZBTB5	-10.563	1.69E-04	7.08E-03
LCN3	-10.697	1.70E-04	7.11E-03
LGALS4	-10.831	1.70E-04	7.13E-03
CXCL4	-10.966	1.71E-04	7.16E-03
CREG3	-11.100	1.72E-04	7.19E-03
LYST	-11.234	1.73E-04	7.21E-03

Supplementary Table 2.

Differential Expressed genes in PBMCs

geneName	betaValue	pValue	qValue
IGHG1	5.076	1.27E-11	1.73E-08
SKA3	4.394	1.69E-08	8.33E-06
BIRC5	4.294	7.58E-14	2.47E-10
SDC1	4.252	1.13E-08	6.17E-06
RRM2	4.105	3.28E-11	3.57E-08
CDT1	4.090	1.34E-10	1.21E-07
IQGAP3	4.062	2.56E-11	2.98E-08
CDC20	4.060	5.07E-16	4.01E-12
MYBL2	4.025	7.38E-16	4.01E-12
TYMS	4.004	1.15E-18	1.88E-14
TK1	3.873	1.15E-15	4.69E-12
TROAP	3.868	4.98E-12	7.38E-09
CCIN	3.847	2.28E-06	6.40E-04
KIFC1	3.786	1.16E-06	3.51E-04
KIFC1	3.786	1.16E-06	3.51E-04
ANLN	3.782	1.18E-05	2.67E-03
CDCA5	3.775	1.33E-12	2.16E-09
UBE2C	3.734	1.21E-13	3.29E-10
PCLAF	3.715	1.22E-10	1.17E-07
IGHGP	3.643	4.94E-05	8.18E-03
CDC25A	3.639	1.79E-09	1.22E-06
CENPA	3.637	2.91E-09	1.90E-06
CDC45	3.634	6.62E-10	4.90E-07
AURKB	3.599	1.74E-13	3.55E-10
RNASE1	3.544	1.55E-05	3.29E-03
PLK1	3.439	2.83E-10	2.31E-07
KIF4A	3.414	4.96E-05	8.18E-03
TUBB3	3.379	2.72E-06	7.53E-04
KIF18B	3.318	1.75E-10	1.51E-07
GALNT14	3.310	1.06E-06	3.46E-04
SPC24	3.293	5.09E-09	3.19E-06
TICRR	3.282	2.50E-08	1.20E-05
CCNB2	3.213	2.35E-07	9.34E-05
HJURP	3.206	3.81E-07	1.41E-04
KIF20A	3.191	3.04E-05	5.77E-03
PKMYT1	3.188	3.26E-10	2.53E-07
ILDR2	3.168	6.92E-06	1.64E-03
KIF2C	3.097	1.72E-07	7.19E-05

GINS2	3.094	1.17E-07	5.16E-05
KCNN3	3.077	3.98E-06	1.08E-03
RAD51	3.077	1.06E-08	5.99E-06
CDCA8	3.062	6.27E-09	3.65E-06
IGHV3-49	3.034	6.43E-06	1.54E-03
TPX2	3.028	1.61E-05	3.37E-03
OIP5	3.026	2.02E-06	5.79E-04
ORC1	3.016	5.30E-09	3.20E-06
GTSE1	3.009	1.53E-07	6.58E-05
NCAPH	2.999	2.05E-07	8.37E-05
LAMC1	2.997	1.72E-06	5.00E-04
ZWINT	2.977	1.58E-09	1.12E-06
CDCA3	2.937	1.24E-08	6.32E-06
ESPL1	2.925	3.22E-07	1.22E-04
UHRF1	2.896	2.14E-11	2.68E-08
HASPIN	2.841	6.99E-07	2.47E-04
IGKV1-12	2.815	6.22E-05	9.94E-03
GAPDHP72	2.786	5.31E-05	8.66E-03
CCNB1	2.769	1.93E-05	3.88E-03
DDN	2.753	4.24E-05	7.60E-03
PYCR1	2.648	1.09E-06	3.47E-04
TEDC2	2.630	1.53E-05	3.29E-03
E2F1	2.621	2.90E-13	5.25E-10
E2F2	2.592	3.94E-11	4.02E-08
SLCO4A1	2.578	2.10E-05	4.18E-03
CENPM	2.562	2.67E-07	1.04E-04
MCM2	2.529	5.16E-08	2.41E-05
KCNK5	2.465	4.13E-06	1.10E-03
HMGB3	2.439	3.99E-05	7.23E-03
IGHV4-34	2.408	3.31E-05	6.20E-03
RGS16	2.382	3.92E-05	7.18E-03
FOXM1	2.381	1.19E-08	6.27E-06
CENPW	2.379	4.82E-05	8.10E-03
ASF1B	2.288	6.28E-06	1.53E-03
POC1A	2.193	7.54E-07	2.56E-04
HIST1H1C	2.139	1.54E-05	3.29E-03
WDR62	1.984	1.23E-05	2.75E-03
PTTG1	1.943	1.92E-05	3.88E-03
UBE2S	1.924	1.55E-06	4.58E-04
CD59	1.872	7.59E-08	3.44E-05
SCD	1.845	1.15E-06	3.51E-04
SLC1A4	1.719	3.70E-05	6.86E-03

TUBB4B	1.683	7.99E-06	1.86E-03
TUBA1B	1.661	4.73E-05	8.04E-03
TUBA1C	1.642	2.27E-05	4.46E-03
TIMELESS	1.616	5.80E-06	1.43E-03
DHCR24	1.580	4.95E-06	1.28E-03
JPT1	1.414	4.36E-05	7.65E-03
LTK	-1.557	2.72E-05	5.22E-03
SEMA4C	-1.712	1.12E-05	2.57E-03
CRIP2	-2.025	4.65E-05	7.97E-03
MS4A7	-2.038	5.93E-05	9.58E-03
RORC	-2.230	4.60E-06	1.21E-03
IGFBP3	-2.539	2.52E-05	4.90E-03
NT5E	-2.548	4.62E-05	7.97E-03
CD1C	-2.599	9.89E-07	3.29E-04
SIGLEC20P	-2.866	1.43E-05	3.15E-03
FCER1A	-2.919	4.45E-07	1.61E-04
KNDC1	-3.169	5.50E-06	1.38E-03
CD207	-3.385	1.75E-05	3.60E-03
CROCC2	-3.882	1.65E-13	3.55E-10
CLEC4F	-4.066	4.30E-05	7.61E-03
DCANP1	-4.184	5.25E-06	1.34E-03
LYPD2	-5.543	7.11E-07	2.47E-04
